# Lyophilization of Liposomal Formulations: Still Necessary, Still Challenging

**DOI:** 10.3390/pharmaceutics10030139

**Published:** 2018-08-28

**Authors:** Silvia Franzé, Francesca Selmin, Elena Samaritani, Paola Minghetti, Francesco Cilurzo

**Affiliations:** Department of Pharmaceutical Sciences, Università degli Studi di Milano, via G. Colombo 71, Milano 20133, Italy; silvia.franze@unimi.it (S.F.); francesca.selmin@unimi.it (F.S.); elena.samaritani@studenti.unimi.it (E.S.); paola.minghetti@unimi.it (P.M.)

**Keywords:** cake appearance, cryoprotectant, freeze-drying, freezing rate, time and temperature, liposomes, lyoprotectant, QbD, solvent, stability, sublimation

## Abstract

Nowadays, the freeze-drying of liposome dispersions is still necessary to provide a solid dosage form intended for different routes of administration (i.e., parenteral, oral, nasal and/or pulmonary). However, after decades of studies the optimization of process conditions remains still challenging since the freezing and the dehydration destabilize the vesicle organization with the concomitant drug leakage. Starting from the thermal properties of phospholipids, this work reviews the main formulation and process parameters which can guarantee a product with suitable characteristics and increase the efficiency of the manufacturing process. In particular, an overview of the cryo- and/or lyo-protective mechanisms of several excipients and the possible use of co-solvent mixtures is provided. Attention is also focused on the imaging methods recently proposed to characterize the appearance of freeze-dried products and liposome dispersions upon reconstitution. The combination of such data would allow a better knowledge of the factors causing inter-vials variability in the attempt to improve the quality of the final medicinal product.

## 1. Introduction

Over the past decades, liposomes have evolved from models for artificial cells due to their structural similarity to biological membranes, to very attractive drug delivery systems. Their use in the pharmaceutical field is in large part due to good biocompatibility [1] and the ability to encapsulate both hydrophilic and hydrophobic drugs in the aqueous core and in the bilayer, respectively [2]. 

The possible limitations in the development of liposomal dosage forms are usually related to phenomena of oxidation and/or hydrolysis of lipids, drug leakage, the formation of aggregates or vesicle fusion, with consequent alteration of the in vivo biodistribution and, therefore, efficacy and safety [1,2,3]. When formulative approaches (i.e., use of saturated and/or high transition phase lipids, addition of antioxidant agents, design of charged and functionalized particles, etc.) or precautionary storage conditions (conservation in inert atmosphere, at −20 °C, protected from light) are not sufficient, the only option to overcome these instability issues is to dry the liposome-based formulations, since all the above mentioned phenomena are facilitated in an aqueous environment. Whenever liposomes are intended to be administered by oral and/or pulmonary routes, the drying of the colloidal suspension is unavoidable [4,5]. Among the possible approaches, lyophilization still remains the main studied technique even if examples of marketed lyophilized drug products are very limited [6]. This is due to the complexity of the process since the choice of excipients and process parameters to protect the membrane integrity from stresses due to freezing and dehydration is challenging. In fact, liposomes contain water and spontaneously assemble in the presence of water, then its removal may cause a significant, sometimes irreversible, alteration of their structure as clearly stated by Chen and co-workers [7].

This work aims to review the literature not only on the formulation and process parameters, which have been demonstrated to stabilize liposomal suspensions, but also on the methods suitable for the characterization of freeze-dried and reconstituted liposome formulations. Moreover, considering that the lyophilization is defined as a highly specialized process known to be complex [8] and that liposomes are considered among the complex formulations [9,10], attention is also given to the quality by design approach and to the related controls.

## 2. Thermotropic Behavior of Phospholipid Bilayers

**Figure 1:**
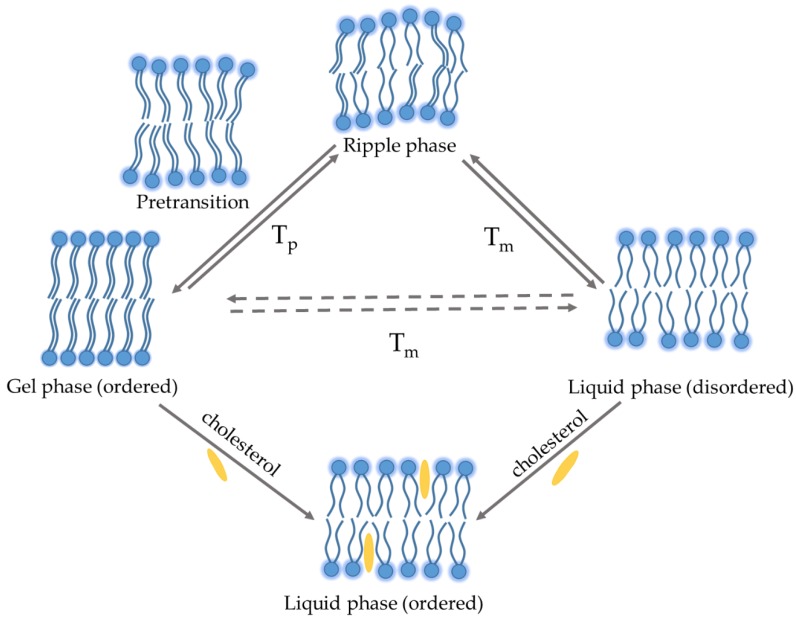
Thermotropic behavior of phospholipid bilayer in an aqueous medium. *T_p_*: pre-transition temperature, *T_m_*: phase transition temperature.

Hydrated phospholipid membranes change between different phases, characterized by different positions (i.e., lateral order) and orientation (i.e., rotational order). The two extreme phases of bilayer’s phospholipids are the so-called gel and fluid phases [11]. The gel phase, also known as solid-ordered phase, is characterized by the formation of a very compact bilayer with a minimum mobility since hydrocarbon chains display an all-trans configuration favoring their maximum elongation (Figure 1).

Above a certain temperature, called phase transition temperature (*T_m_*), the lipid bilayer undergoes a gel–liquid transition: the lipid chains change configuration (from all-trans to all-gauche) and assume a less extended and compact structure. Depending on the chemical structure (i.e., length and saturation degree of the acyl chains, nature of the polar heads, type and ionic force of the dispersion medium) different lipids can have dramatically different *T_m_* (Table 1).

In particular, the length and degree of unsaturation of the acyl chains have the most prominent effect [13,16]. The longer the length of the acyl chains (or the lower the degree of unsaturation), the higher the *T_m_* of the phospholipid (Table 1). In some cases, the transition from gel to liquid phase is preceded by a gradual change in the distance among polar heads (pre-transition, *T_p_*) occurring a few degrees before *T_m_* (~5–7 °C). Above the *T_p_* value a simultaneous variation in the phospholipid configuration and membrane curvature occurs so that the bilayer surface is characterized by periodic one-dimensional undulations, called the ripple phase (Figure 1). It is assumed that ripples are caused by the alternation of gel and liquid lipid domains in a single monolayer: as different domains have different geometrical characteristics, lipids are forced to arrange on the surface [17]. The difference between *T_p_* and *T_m_* depends again on the length of hydrocarbon chains: the longer the length, the lower the difference, up to the complete overlap for acyl chains having 22 carbon atoms [17,18]. Also in the case of mixed chains (i.e., egg phosphocholine (EPC)) with a prevalence of C_18_ component, *T_p_* moves towards *T_m_* and this, along with the presence of different metaphases, does not allow to discriminate the two *T_m_* values (Table 1). On the other hand, it is reported that saturated acyl chains ranging between C_10_ and C_13_ do not pass through the ripple phase, but they undergo a direct gel-liquid transition (Table 1) [13].

In addition, depending on the hydration level, hydrocarbon chains can be tilted or not tilted; the angle of tilt increases with the increase of water content, obtaining a thin bilayer [19]. Other factors, such as the type of phospholipid polar head or the presence of cholesterol, may influence the chain tilt [20]. Cholesterol is one of the main components of liposomal formulation along with phospholipids, since it regulates the fluidity of the bilayer and stabilize the membrane. As a matter of fact, the hydrophobic steroidal moiety of cholesterol (and other similar substances) promotes the all-trans configuration of acyl chains decreasing the tilt angle in gel phase. Moreover, the incorporation of cholesterol in the bilayers causes a broadening, or elimination, of the *T_m_* because it exerts an ordering effect on the liquid phase that assumes physical characteristics in common with the solid-ordered gel phase. In fact, the bilayer configuration in presence of cholesterol is defined liquid-ordered phase [21]. 

**Figure 2:**
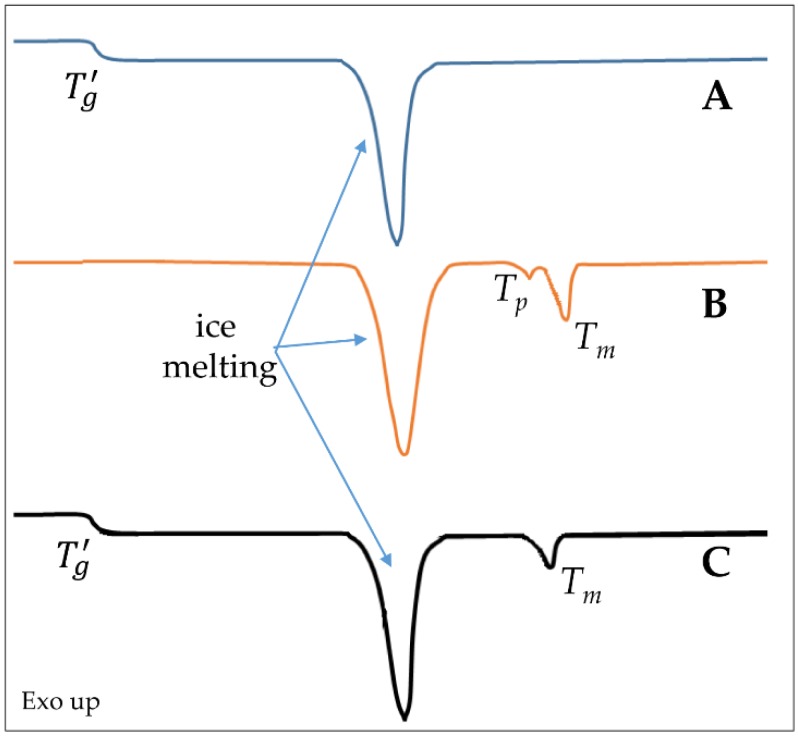
Schematic representation of thermal events detectable by differential scanning calorimetry (DSC) in a simplified system: (**A**) typical trace of an amorphous protectant; (**B**) typical pattern of liposomes made of a phospholipid with a *T_m_* significantly higher than melting ice temperature; (**C**) representation of an ideal thermogram registered on a liposome dispersion containing the protectant. *T_g_’:* glass transition temperature of maximum freeze concentrate; *T_p_*: pre-transition temperature of fully hydrated phospholipids; *T_m_*: phase transition temperature of fully hydrated phospholipids.

Differential scanning calorimetry (DSC) and microcalorimetry analyses are often used to study the response of the lipid membrane to formulation additives and/or external factors, being either a drug or an environmental stress. Nevertheless, these techniques can be applied only on very simplified formulations, since for example the presence of cholesterol significantly decreases the enthalpy variation associated *T_m_* and, at high concentrations, hinders the transition [22]. Furthermore, lipids with *T_m_* values close to the fusion of ice have to be generally replaced by other lipids (mainly 1,2-dipalmitoyl-sn-glycero-3-phosphocholine (DPPC), Table 1) to observe the thermic events in a proper manner [23]. A typical DSC pattern of lipid vesicle aqueous dispersion is represented in Figure 2. An alternative technique is the fluorescence spectroscopy that can permit the measurement of the dependence of the membrane fluidity on the variation of temperature also in presence of cholesterol [24]. Furthermore, small-angle X-ray scattering (SAXS) or electron spin resonance [25], along with ellipsometric techniques [26] and Raman spectroscopy [24], can provide useful information on the structural changes of the bilayers over a broad range of conditions.

## 3. Stresses of Liposomal Dispersions Occurring During Freeze-Drying 

A typical freeze-drying process consists of three main phases: freezing, primary drying and secondary drying. The freezing phase is a cooling step where most of the solvent is separated from the liposomes and excipients, resulting in the formation of ice crystals. The annealing of a frozen sample can be also introduced to decrease the sample heterogeneity and reduce the drying rate mainly due to the increase of ice crystal size. The primary drying is initiated when the chamber pressure is reduced to a few millibars and the shelf temperature is increased to supply heat required for sublimation. During the secondary drying, temperature is raised to allow the water desorption. All these steps may be critical for the liposome integrity and the retention of the entrapped compounds.

### 3.1. Freezing Step

The freezing step can induce many destabilizing stress factors because *T_m_* is related to the hydration state of phospholipids, and liposomal dispersions can account for different types of water pools; bulk water and intraliposomal solution freeze at approximately −20 °C (heterogeneous ice nucleation) and −45 °C (homogeneous ice nucleation), respectively [27,28]. 

**Figure 3:**
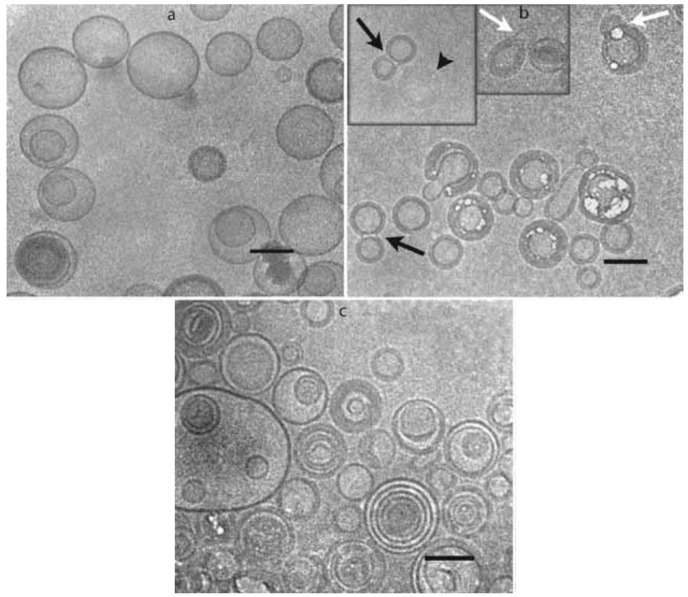
Cryo-transmission electron microphotographs of 1,2-distearoyl-sn-glycero-3-phosphocholine (DSPC)/cholesterol (60:40, mol/mol) liposomes prepared in water (**a**) and after addition of 10% (*w*/*v*) lactose to the external solution (**b**). The microphotograph shown in (**c**) was collected after freezing the liposome dispersion shown in (b). The black arrows denote peanut shapes structures, white arrows collapsed outer membrane of double liposomes and arrowhead in the inset of (**b**) denotes a completely collapsed liposome. Bars 1⁄4 100 nm. Reproduced with permission from [31], Elsevier Inc., 2010.

At high hydrations and above freezing temperatures, lipid–water suspensions separate into two different phases: a lamellar phase with about 30 water molecules per lipid and a bulk phase of nearly pure water [29]. Decreasing the temperature, the bulk water starts to freeze and liposomes approach to each other closely. Then, the lamellar phase begins to dehydrate decreasing the spacing between the phospholipid head groups. These events lead to a lateral expansion of the bilayer resulting in a compressive stress in their plane. The possible consequences depend on the bilayer composition and include demixing of more than one component in vesicles, micelle formation and aggregation [29]. Moreover, the freezing determines a cryoconcentration of the solutes (i.e., excipients) in the bulk solution, creating an osmotic gradient which causes a loss of the internal solution with consequent leakage of dissolved hydrophilic drugs [30]. As an example, the addition of 10% of lactose to 1,2-distearoyl-sn-glycero-3-phosphocholine (DSPC)/cholesterol liposomes caused the reduction of internal volume and simultaneous invagination of the bilayer and self-fusion events which lead to peanut-shaped liposomes (Figure 3) [31]. The osmotic shock is described to be independent of the liposome size [32].

Freezing rate also has a significant impact on the maintenance of liposome structure. Ultrafast cooling (e.g., immersion of small volume suspensions in liquid nitrogen) results in the formation of fine ice crystals and a homogeneous distribution of the protectant, which might reduce the disruption of the liposomal bilayer structure. On the other hand, a slow freezing rate reduces the supercooling [27] and the osmotic pressure since water molecules can diffuse slowly across the bilayer when the external solution becomes freeze-concentrated [33]. Consequently, a slow freezing rate (lower than 0.5 K/min) may minimize the formation of ice crystals in the inner aqueous compartment and prevent drug leakage [33]. Moreover, it was also hypothesized that slow freezing (1) provides more time to recover from deformations produced by osmotic pressure and mechanical forces; (2) reduces the vesicles located at the glass-ice boundary favoring their distribution in the glass matrix; and (3) reduces the stress vectors affecting the rigid bilayers. In general, the effect of the freezing rate strongly depends on the rigidity of the bilayer, or in other words, on lipid composition and presence of cholesterol. As an example, DPPC liposomes are more stable than EPC ones during freezing (Table 1). Furthermore, liposomes containing cholesterol in the bilayer are less damaged from a quick cooling because of the increased order of the fluid phase in the presence of cholesterol [34].

The annealing of a frozen liposomal dispersion, known to improve the cake appearance, caused the drug leakage of a water soluble-encapsulated drug probably because the bilayer underwent a phase change [35]. Alternatively, two isothermal steps at 5 and −5 °C [36,37] can be added to reduce the intrinsic stochasticity in this step.

### 3.2. Drying Steps

The possible detrimental effects of drying steps mainly emerge during the rehydration of the product [33]. As a matter of fact, the spatially separation of phospholipids is exerted by the water molecules bound to the polar heads of lipids. The dehydration of phospholipids favors the hydrophobic interactions between the acyl chains increasing the packing density of the bilayer [38]. As a result, the bilayer moves from a hexagonal phase, in which the lipid head groups surround channels of water, to a ribbon phase in which lipid bilayers are packed to form a two-dimensional lattice [39]. The increased order of the bilayer, demonstrated also by the decrease of the tilt of the hydrocarbon backbone following dehydration, causes a drastic increase of the *T_m_* (up to 60 °C) [38,40]. This increment seems to be strictly dependent on the nature of the polar heads of lipids, or, in other words, on the type and the entity of the interactions occurring between polar heads and water molecules and/or between adjacent polar heads. As an example, the strong intermolecular interactions occurring between the ammonium and phosphate groups in phosphatidylethanolamines causes a drastic increase of *T_m_* after dehydration (from 63 to 100 °C) [40]. Thus, the stabilization of liposomal structure relies on the maintenance of the *T_m_* at the values of the fully hydrated bilayers to avoid a gel–liquid transition during reconstitution since the rehydration of phospholipids is associated to drug leakage. This goal may be obtained pre-heating the sample in the dry phase above the *T_m_* or using excipients interacting with the polar regions of liposomes able to maintain the liquid crystalline state [41]. It should be mentioned that cholesterol itself may have a protective effect on the drying of liposomes. In fact, as stated above, cholesterol causes a decrease in the *T_m_* reducing the interactions among the acyl chains. Moreover, it may interact with the polar heads of lipids through H-bond formation due to the exhibition of the −OH group on the interfacial region. Indeed, some authors hypothesized a possible competition between cholesterol and protectant excipients in the interaction with lipid polar heads [42]. As a matter of fact, it was observed that the drug leakage upon rehydration is lower in the presence of cholesterol [35].

From a process point of view, the primary drying can be considered a fine balance between mass and heat transfer since the rate of the mass transfer is proportional to the latent heat of sublimation of ice per unit mass. Moreover, the energy absorbed during sublimation must be compensated by a supply of energy from another source, e.g., shelf, to avoid a dramatic reduction of the drying rate. To define the process parameters of this stage, it is essential to determine the maximum allowable product temperature. In the case of liposomal formulations, this temperature is usually related to the glass transition temperature of maximum freeze concentrate (*T_g_’*). This is a unique value, where the residual non-ice phase forms a glass. In another words, the maximally freeze concentrated solution becomes glass below *T_g_’* reaching viscosity values to the order of 10^13–14^ P; whereas increasing the temperature above *T_g_’* results in an increase in mobility and the system changes to rubbery or liquid phase. This transition can occur over a broad temperature range [43].

**Figure 4:**
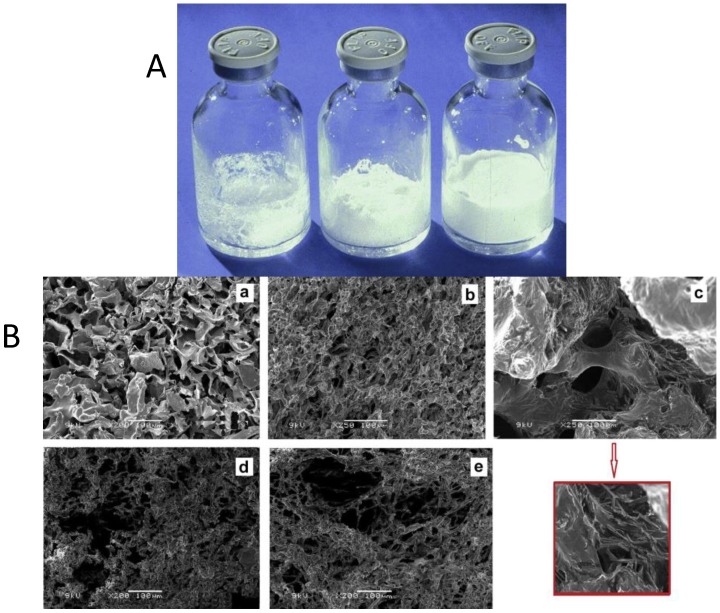
Possible macro and micro appearance of cakes obtained by freeze drying. (**A**) Collapsed cake: total collapse (left) and partial collapse (center). The vial on the right shows no evidence of collapse; (**B**) SEM images of freeze-dried liposomes in presence of trehalose (S:L = 5:1) with a 200 times magnification (**a**); in presence of sucrose (S:L = 5:1) with a 250 times magnification (**b**); in presence of sucrose and mannitol (S:L = 5:1) with a 250 times magnification; no lyoprotectant (control) with a 200 times magnification (**d**); in presence of trehalose (S:L = 3:1) with a 200 times magnification, (**e**) respectively. S:L represents the carbohydrate to lipid molar ratio. The insert in red highlights the crystallized mannitol in sample (**c**). Reproduced with permission from [50,51], Elsevier Inc., 2018.

*T_g_’* values are considered predictive of the collapse temperature (*T_c_*), namely the temperature at which the frozen product can no longer support its structure and structural modifications, e.g., holes or fissures, may be observed. If the sample temperature rises above *T_c_* during the primary drying, the cake can collapse (Figure 4A) with the concomitant vesicle fusion and/or aggregation [44,45]. Thus, a true measure of *T_c_* is desirable to avoid a time (and energy) consuming process. To this end, light transmission freeze-drying microscopy (FDM) is usually used. Alternatively, a new type of freeze-drying microscope based upon time-domain optical coherence tomography (OCT-FDM), recently proposed to design a lyophilization process [46,47], can be also used since it was able to detect formulation at low solid content, as in the case of most of the freeze-dried liposomal formulations. 

The secondary drying, or desorption step, determines the residual moisture content in the final freeze-dried product and, therefore, its long-term stability and the quality of the reconstituted pharmaceutical dosage form. The water content which cannot be removed by sublimation (*W_g_’*) can be considered the most significant factor determining the operative conditions of this step. As a matter of fact, *W_g_’* is the content of plasticizing water in a glass matrix and, since the greater the *W_g_’* the lower the glass transition (*T_g_)* value of dried product, it determines the long-term stability of the product [48]. This feature also implies that not only the process, but all factors causing a variation of moisture content during storage, e.g., stoppers [49], should be carefully evaluated.

Moreover, the secondary drying can cause overheating when the heating ramp or the holding time are not carefully investigated. Only recently, the use of Raman and near-infrared (NIR) spectroscopy was proposed as a complementary tool of Pirani measurements to supply the information about the acyl chain packing of the liposome bilayers and predict if any harmful events would occur. This approach would allow, giving a better understanding of the process, to improve process efficiency by optimizing the primary and secondary drying time [50].

## 4. Stabilization by Excipients

Excipients with different biophysical and chemical properties, namely the protectants, are included in outer aqueous phase of liposomal dispersions to stabilize the liposome membrane during freeze-drying and reduce the detrimental effects, namely alterations of physical features of vesicles and/or drug leakage, caused by the dehydration of phospholipids and cryo-concentration. Generally speaking, amorphous protectants are preferred to crystalline substances which can damage phospholipid bilayer during freezing [52]. 

Sugars, and particularly disaccharides, are considered excipients of choice, although after decades of studies on liposomes the protection mechanism is not fully understood. In 1973, Crowe et al. proposed the water replacement hypothesis: the interactions, namely H-bonds, between the carbohydrates and phospholipid head groups replace those with water, stabilizing the vesicle structure [53]. Later, it was demonstrated that sugars can bridge three phospholipids simultaneously through multiple H-bonds [54]. More specifically, these excipients can simultaneously link through H-bond to the carbonyl and phosphate groups of the polar heads and/or to the methyl group of the hydrophobic moiety. Nevertheless, the phosphate group is the favorite for the interactions [55]. Fourier transform infrared (FTIR) spectroscopy supported the formation of these interactions since the bands attributed to phosphate, carbonyl and ammonium moieties shifted toward other frequencies after the freeze-drying cycle [53].

**Figure 5:**
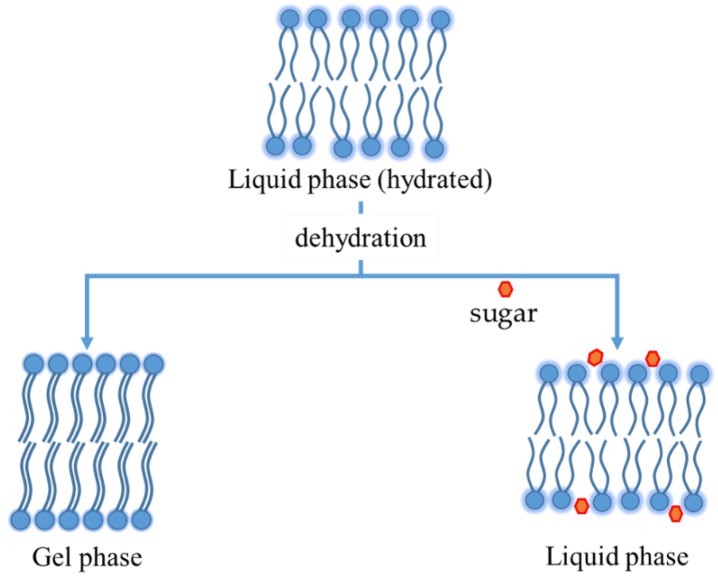
Phospholipids bilayer before and after dehydration, with and without sugar.

This behavior explains why the bilayer *T_m_* decreases when a disaccharide is added. Sugars increase the distance between phospholipid polar heads, minimizing van del Waals interactions among hydrocarbon chains (Figure 5). As a result, sugars not only limit the *T_m_* increasing during dehydration, but they can also decrease *T_m_* in completely hydrated bilayers (as schematized in Figure 2). The interaction seems maximized for a sugar:lipid mass ratio of 5:1 [56], even if in the case of disaccharides a beneficial effect occurred also at lower ratios (Table 2). As an example, when sucrose was added to paclitaxel loaded pegylated liposomes, the paclitaxel retention was guaranteed already at a sucrose concentration of 150 mM (sugar:lipid ratio 3:1 w/w) but in preventing the aggregation assuring the presence of a monodisperse population after lyophilization, the maximum protectant effect was observed at 5:1 sugar: lipid ratio (Table 2) [56]. Instead, in the case of monosaccharides, such as glucose, a higher ratio (at least 9:1) may be required to have enough compound interacting with the polar heads of lipids [57]. The concomitant encapsulation of a protectant in the aqueous core of the vesicles can be beneficial because it abolishes, or at least reduces, the osmotic gradient caused by cryoconcentration, preserving the size. Accordingly, it was demonstrated that the stabilization effect of disaccharides is optimized by adding the excipient both inside and outside of liposomes with a 2:5 internal to external ratio [56].

The protective effect of sugars can be further explained considering the vitrification model. According to this theory, upon freezing sugars form an amorphous phase with high viscosity and low mobility that acts as a barrier between adjacent bilayers. This glassy matrix limits the fusion of vesicles and protects the bilayer from damages caused by ice formation [58]. In other words, sugar molecules in the bulk solution permit to maintain the distance among vesicles. As a result, sugars avoid the increase of *T_m_* and the leakage of hydrophilic compounds induced by the detrimental effect of extra-liposomal ice (see above). To corroborate the implication of the vitrification theory in the protection of liposomes by sugars, it was assumed that the surface tension of vesicles decreases because of the formation of interactions among the liposome surface and the glassy matrix [59].

It is well-accepted that water replacement and vitrification theories are not excluding each other, but rather these mechanisms cooperate to protect liposomes during freeze-drying [7]. However, they cannot completely explain the results reported in the literature. Indeed, it should be also considered that the *T_m_* of dispersions increases as the water activity decreases in the presence of high concentrated solutes [68]. As an example, Strauss et al. found that the addition of up to 10% sucrose to hydrated multilamellar vesicles of DPPC elevated the *T_m_* by several degrees [69]. Furthermore, the thermal behavior of the system is influenced by the type of vesicles and the extent of protectant. In the case of large DPPC multilamellar liposomes, the addition of mono- and disaccharides raised and broadened the *T_m_*. The addition of high strengths of sucrose and trehalose to unilamellar vesicles made of the same phospholipid created multiple metaphases [70]. Furthermore, it is generally recognized that high quantities of sugars, that are required to guarantee a reproducible protective effect, could influence the viscosity and, therefore, the rehydration of the vesicles [71]. In the definition of the amount of protectant, its impact on the tonicity of the reconstituted solution has to be carefully evaluated to avoid a negative effect on the safety of the drug product administered by ocular and parenteral route. However, this aspect is not sufficiently discussed in the literature.

The use of more complex carbohydrates gave contrasting results. As an example, maltodextrins are commonly used to preserve the main of nanomedicines upon lyophilization [72], since *T_c_* increases with the increase of glucoside units. In other words, in theory, maltodextrin or dextran would be more convenient to be freeze-dried with respect to trehalose and sucrose (Table 3). However, increasing the glucoside unit number the steric hindrance increases and number of H-bonds among the sugar and the polar head decreases, limiting the stabilizing effect (Table 2) [62]. Similarly, hydroxyethyl starch, although the high *T_g_* that allows to prevent the vesicle fusion, had a poor stabilization effect [61]. In fact, it was not able to depress the *T_m_* in the dehydrate state and, therefore, it could not prevent the leakage of the entrapped drug upon rehydration of the liposomes (Table 2).

Instead, dextrans with a molecular weight up to 40 KDa avoided the aggregation of lipoplexes after lyophilization [63]. Dextran at higher molecular weight (480 KDa) was able to stabilize DPPC liposomes, but completely failed in the case of liposomes composed of unsaturated lipids having very low *T_m_* such as EPC (Table 2) [73]. In the last case, the lack of stabilizing effect was ascribed to the fact that dextran was not able to depress the *T_m_* and/or interact with polar heads, inhibiting by a non-competitive mechanism also the effect of trehalose. Other polysaccharides have been also tested. As an example, hydroxypropyl methylcellulose (HPMC) 4000 in combination with mannitol resulted effective to preserve the main physico-chemical features of felodipine loaded liposomes intended for cutaneous application [74].

Liposomes to be given parenterally are generally functionalized with hydrophilic moieties, mainly poly(ethylene glycol) (PEG) 2000, to reduce the opsonization phenomenon, or with targeting agents able to drive the carrier to particular cells or tissue. Hyaluronan (HA) is one of the most studied targeting agents, for example, in anticancer therapy [76]. Peer and coworkers suggested that the decoration of liposomes with HA may also protect liposomes upon lyophilization [71]. As far as PEGylated liposomes is concerned, it should be mentioned that the freeze-drying of these formulations has some additional challenges [77]. In fact, owing to instrumental limitations, the process temperatures are generally above the *T_g_’* of PEGs which range from −85 to −65.8 °C, depending on the molecular weight [63]. The selection of protectants has to be carried out with the purpose to increase the *T_c_* value of the whole system and, at the same time, considering the compatibility of the selected excipient with PEG [7]. As an example, dextrans with a molecular weight higher than 5 KDa were not compatible with PEG causing liposome aggregation also at very low temperatures of storage (i.e., −20 °C) [63]. Instead, the cyclic oligosaccharide hydroxypropyl-β-cyclodextrin (HPβCD) allowed the reconstitution of the formulations and resulted more effective in avoiding the drug leakage than trehalose and sucrose probably due to the huge number of hydrogen donors and acceptors present in the molecule [65]. The protective effect of HPβCD was ascribed to interactions with the polar heads of phospholipids that impact on the packing of the hydrophobic moieties. However, the presence of cholesterol and the saturation of the phospholipid acyl chains significantly influenced the HPβCD performances [24].

Recently, Popova et al. proposed glycosides as novel excipients able to protect the lipid membrane. With respect to the classic carbohydrates, flavonols are amphipatic compounds enable both to form H-bonds with the polar heads of lipids and to intercalate in the liposome bilayer. The stabilizing effect, however, resulted to be strictly dependent on the nature of flavonols (i.e., the extent of glycosylated moieties) and on the composition of the vesicles. In particular, it was hypothesized that the presence of non-bilayer lipids favored the localization of the flavonols on the bilayer surface, leading to the stabilizing effect [78].

Beside sugars, other molecules with different protection mechanisms were investigated as adjuvants for freeze-dried liposomes. They include also amino acids: proline, arginine, lysine and histidine [44]. Amino acids, used with disaccharides, can stabilize the system not only with H-bonds, but also with electrostatic interaction. For this purpose, lysine seems to have similar protective effects to trehalose [44]. Moving the attention to proteins, the loading of gelatin inside the vesicles permitted the formation of a gel during the cooling of the liposomal dispersion providing an inner mechanical support [67] and preventing the drug leakage [79]. Later studies of the same research team highlighted that by covering the external surface of liposomes with polymers (in this study calcium alginate), their physical stability upon freeze-drying increased [80].

It should be mentioned that, even if protectants are essential to allow the reconstitution of liposomal formulations, their *T_g_’* values are generally lower than *T_c_* (Table 3) and, therefore, their use in the definition of primary drying protocols leads to conservative cycle. In the same way in the selection of a protectant, its *T_c_* or *T_g_’* value should be taken into consideration since the lower the value the shorter the freeze-drying cycle. 

### Use of Organic Solvent as Adjuvants

Organic solvents, usually tert-butyl alcohol (TBA), are widely used to increase the sublimation rate (which allows to design shorter processes), the chemical stability of pre-dried bulk solution and dried products [81]. 

In the attempt to increase the efficiency of the manufacturing process, the possibility of freeze-drying already formed liposomes using a TBA/water co-solvent system was investigated. Although TBA is not monographed in the United State Pharmacopeia (USP) or European Pharmacopeia (EP), neither listed in the guideline on impurities issued by the International Council for Harmonisation of Technical Requirements for Pharmaceuticals for Human Use (ICH), it is likely to fall in the category of a class 2 solvent based on the similarity of acute DL_50_ toxicity data [82]. The addition of low concentration of TBA (<20%) results in the crystallization of bulk ice and eutectic A [83]. Moreover, in the presence of TBA, needle-shape crystals were formed with a substantially larger surface area that the spherical ice crystal formed when TBA was not present [84]. The higher specific area, the faster the sublimation of solvents and, therefore, the shorter the primary drying stage. However, the eutectic A crystals would cause extra injuries in liposomes, as in case of soybean phosphatidylcholine liposomes [85] or a reduction of the entrapment efficiency after freeze-drying [86]. This tendency can be counterbalanced by the presence of a protectant, such as sucrose, which can significantly inhibit the crystallization of the eutectic A [85]. However, the addition of solutes characterized by a high *W_g_’* value can affect also the residual TBA in the dried product [87]. Indeed, solvents can be retained within the freeze-dried cake after lyophilization. Two theories used to explain this phenomenon are “selective diffusion” and “microregion entrapment”. It is postulated that the retention is not due to adsorption to the dried material. The solvent retention appears to occur in localized micro-regions where the solvent is initially frozen, and the exacerbation of secondary drying conditions do little decrease the residual amount probably because of the formation of H-bonds to the dried product [81].

## 5. Quality by Design

To obtain a well-design freeze-drying process, the application of Quality by Design (QbD) paradigm should be also applied based on the following aspects [88]:

1. definition of the target product quality profile,
2. determination of the critical quality attributes (CQAs) and critical process parameters (CPPs),
3. risk assessment,
4. development of an experimental design aiming to investigate the impact of CPPs on CQAs and establish a design space
5. design and implementation of a control strategy to ensure a continuous improvement.

**Figure 6:**
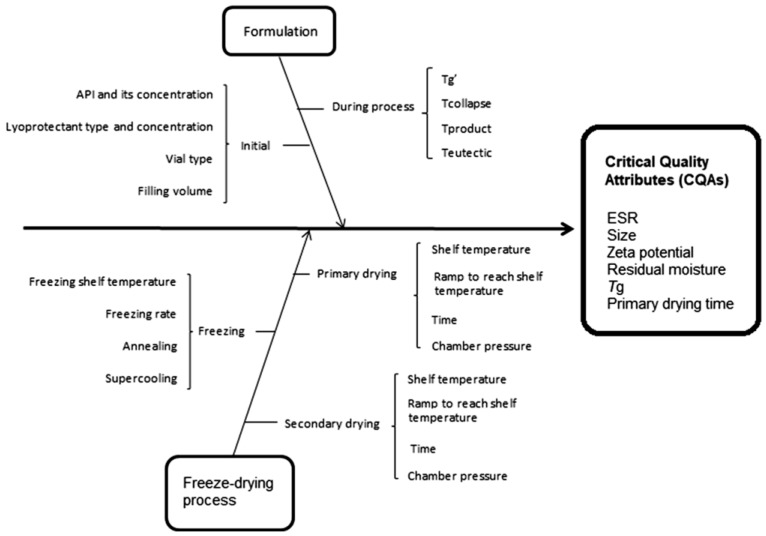
Ishikawa diagram illustrating factors that have impact on the quality attributes of the final product. Reproduced with permission from [89], Informa UK Limited, 2017.

As an example, Sylvester et al. applied the QbD approach to improve the final product quality of liposomes loaded by pravastatin in terms of drug leakage, size, *ζ-*potential, residual moisture contents, *T_g_* and cake appearance, and to shorten the freeze-drying process as much as possible [89]. Among the CPPs influencing the CQAs (Figure 6), the impact of freezing rate, inclusion of an annealing step and primary drying at temperatures below and above *T_g_’* or slightly higher *T_c_* were investigated. 

The results indicated that the process delivers the desired quality of the product as long as operates within the design space. Moreover, the generated model through the D-optimal experimental design could be further used to select an optimal formulation out of the design space. Despite the general knowledge on this topic, the optimal formulation containing both trehalose and mannitol was frozen at 1 °C/min and annealed for 2 h at −25 °C; the primary drying was carried out at −20 °C for about 22 h [89]. This approach can be obviously applied to the other critical factors affecting the quality of lyophilized liposomal formulation, namely, the freezing temperature [90] and the selection of protectant [91].

## 6. Quality Attributes of the Freeze-Dried Liposomes

**Figure 7:**
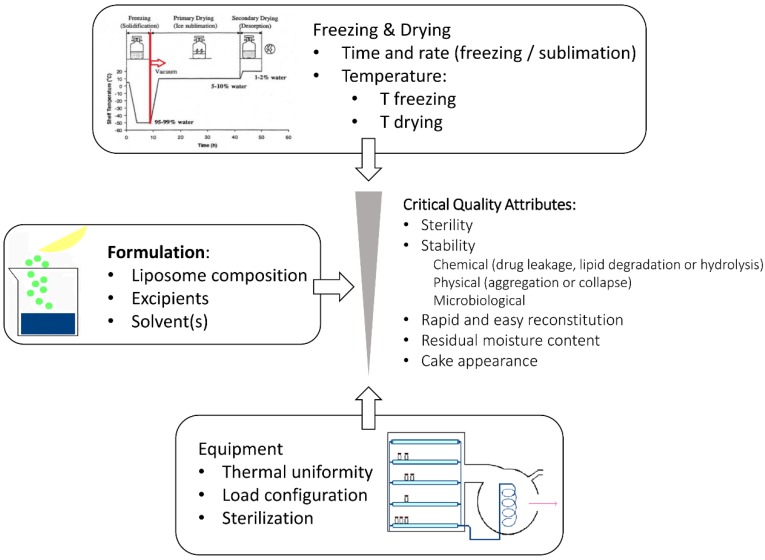
Schematic representation of the factors influencing the critical quality attributes of freeze-dried liposomes.

Liposomes are intended to optimize pharmacokinetic and pharmacodynamic properties of the loaded drug. Consequently, it is mandatory to identify the physical, chemical and biological properties of a liposomal formulation that would influence the in vivo performances and to develop reliable assays aimed to assure that the quality of the formulation is maintained after reconstitution of the freeze-dried product. Thus, two sets of CQAs should be identified: the former defines criticism of the lyophilized products, e.g., the residual moisture content and, when necessary, the cake appearance and the reconstitution time; the latter is referred to the reconstituted, and eventually diluted, liposome dispersion, which represents the final pharmaceutical dosage form (Figure 7). The sterility of the product is an important attribute whenever parenteral, pulmonary or ocular formulations are considered.

### 6.1. Criticism of The Lyophilized Products

The drying processes largely determine the residual moisture content of the final dried liposomal product which in turn affects its long-term stability [92]. As an example, when the residual water content was <1% in freeze-dried doxorubicin-loaded liposomes no significant physical instability or chemical degradation was observed over a 6-month period at temperature up to 30 °C [93]. Furthermore, the residual water content directly influences *T_g_* of the protectants, which is related to the stabilization of liposomes in the glass matrix [27,88]. Indeed, after freeze-drying amorphous components remain vitrified only in case that the storage temperature (*T_s_*) is well below *T_g_* of the glass. As a rule of thumb, a temperature difference “*T_g_* − *T_s_*” of, at least, 50 °C was proposed [93].

The traditional batch-wise lyophilization practice has some important drawbacks mainly due to uncontrolled vial-to-vial and batch-to-batch end product variability. Hence the consistency of some of the CQAs mentioned above cannot be easily achieved as lyophilization is a complex interplay of dynamic and nonequilibrium processes. From a regulatory point of view, such uncontrolled variability, which could lead to unexpected features of a medicinal product, is not desired. Thus, there is a clear need to move in different directions from the enhancement of the process knowledge to the extension of the extensive process control through the definition of techniques to monitor and control the quality attributes of the freeze-dried product in a rapid and non-destructive way. Moreover, the FDA Inspection technical guide on lyophilization of parenterals states that “*the USP points out that it is good pharmaceutical practice to perform 100% inspection of parenteral products. This includes sterile lyophilized powders. Critical aspects would include the presence of correct volume of cake and the cake appearance. With regard to cake appearance, one of the major concerns is meltback*” [94]. Indeed, the examination of the cake appearance is an important attribute of freeze-dried products since it can be observed on the entire batch and harmonized nomenclature and description for variations in cake appearance was recently proposed to overcome challenges related on how to judge the criticality of cake appearance [51]. However, this attribute may or may not be critical with respect to product safety and efficacy. Indeed, sometimes ”a non-ideal cake appearance”, namely not elegant and uniform, has no impact on product quality and it is an inherent characteristic of the product due to formulation, drug product presentation, and freeze-drying process. As an example, a remarkable influence of TBA on final liposomal product was that the cake was friable. Manual shaking led to the formation of free-flowing powder. This quality attribute of dried cakes may result from the modification of the ice habit (i.e., needle-shape vs spherical ice-crystals) in the presence of TBA, but it did not impact on the size distribution of liposome dispersions upon reconstitution [81]. 

Nevertheless, the cake appearance may be also related to changes in the microstructure of the product and, in that case, this quality attribute has an important relevance. For this reason, the visual inspection of the cake is not enough, but it has to be supported by imaging techniques such as scanning electron microscopy (SEM) that allows to analyze the surface topography, roughness, and morphology of solids at high resolution [95]. For example, as stated in the Section 4, the crystallization of the protectant upon lyophilization can damage the liposome structure. This effect may be observed by microscopic evaluation of freeze-dried cakes (Figure 4B). However, there are several criticisms which limit the possibility to obtain quality images by this technique. First, unwanted changes in the freeze-dried product can occur during sample preparation, e.g., during sputter coating and the vacuum during the measurement. Furthermore, due to low optical contrast, the morphology of lyophilized products is difficult to observe, and internal structures are normally not visible, unless the cake is fractured. Because most lyophilized substances are hygroscopic and fragile, a brief contact with the humidity in the atmosphere can alter the cake texture or the product can be damaged upon removal of the from the vial. As an alternative, X-ray microcomputed tomography (μCT) was proposed to provide a wide range of structural information on the surface and cross-sectional morphologies of the scaffolds, including pore distribution, size, and interconnectivity [96,97].

NIR spectroscopy was investigated to offer real-time information on CQAs, such as water to ice conversion, product crystallization, solid-state characterization and residual moisture determination. As already mentioned, NIR spectroscopy was also used as a complementary in-line tool to determine the drying end point in the attempt to optimize of freeze-dried liposomes [89]. However, the inherent limitation of this method is that only a fraction of the vial can be monitored, and intra-vial variability is thus not taken into account [89]. More recently the use of near-infrared chemical imaging (NIR-CI) was proposed for process monitoring because it combines the chemical information from the spectral features in the NIR region with the spatial information on the constituent distribution derived from the pixel-to-pixel spectral variation [98]. NIR-CI allows to discriminate among mannitol-sucrose formulations lyophilized with different drying time, to reveal within-vial inhomogeneity in water content and to characterize the solid-state of the entire lyophilized cake with a high spatial resolution [98]. However, some practicalities are still to be overcome in view of a broader applicability.

Nowadays, the attention is also focused on the study of the compressive mechanical properties of the freeze-dried cakes directly in glass vials by using a texture analyzer [99]. The optimized experimental set-up evidenced that cakes formed by different excipients, namely mannitol, sucrose, treahose [99], gelatin maltodextrin, and poly(vinyl pyrrolidone) (PVP) K40 [100] exhibited different brittle cracking/crushing failure. Furthermore, this technique was sensitive to differences in freezing conditions for sucrose formulations and the moisture content of sucrose cakes [99].

When unsaturated lipids are present in the formulation, the full degradation profile of lipids should be carefully monitored in the liposomal cake, with particular attention on the formation of lysolipids, which are the cause of toxic reactions, such as hemolysis and apoptosis. The lysolipid content has to be controlled both during the production process and the shelf-life of the product [101]. The extent of lipid degradation or peroxidation should be monitored over a range of storage temperatures because the phenomenon is temperature dependent and may increase increasing the storage temperature [102].

### 6.2. Criticisms of The Reconstituted Liposomal Dispersion

The ability of the freeze-dried cake to readily reconstitute upon addition of an appropriate medium is dependent on several factors. Undesired physical structure of the freeze-dried powder (e.g., degree of cake collapses or meltback, the surface area of the cake and the inhomogeneity of the dried matrix) can lead to poor wetting, dispersion and capillarity, agglomeration, unwanted particulate formation during reconstitution, or prolonged reconstitution time. Currently, no procedures are available to monitor the reconstitution of freeze-dried pharmaceutical formulations. Moreover, modalities of reconstitution are also relevant for the generation of the desired average particle size distribution of liposomes avoiding the formation of aggregates which may determine the incidence of undesired reactions. The main findings reported in the literature on this topic are summarized in Table 2.

Destabilization of a reconstituted colloidal suspension can be evaluated by dynamic light scattering (DLS) which is the most user-friendly technique, and it yields relatively accurate and consistent results in a rather short period of time. Differences in size distribution, a polydispersity index close to 1 and high coefficients of variation are indices of fusion and/aggregation. DLS is also known to have several drawbacks, which are mainly inherent to the principles of the technique. Particle size is determined from fluctuations in scattered light intensity due to the Brownian movement of the particles. Since its measurement considers the intensity of light scattered by spherical particles, this technique is not very sensitive for highly polydisperse and non-spherical colloidal systems [103]. FDA guideline indicates that nanoparticle tracking analysis (NTA) can be also used to evaluate size distribution of a liposomal suspension both during research and routine quality controls. Considering that NTA also provides quantitative information [104], the potentiality of this technique can be also exploited to evaluate the concentration of liposomes in a known volume of reconstitution medium.

Along with the particle size, it is important to ensure that the morphology and/or structure of the liposomes do not change after reconstitution. The lamellarity of liposomes in fact impacts on the drug release kinetics, along with the stability of liposomes against protein once injected and, more in general, on their in vivo performances. The structure of the liposome after reconstitution could be examined by imaging analyses usually using transmission electron or cryoelectron microscopy. Moreover, to obtain a deeper insight into the fine structure of the bilayer, SAXS or small angle neutron scattering measurement could be adopted [39].

Generally speaking, liposomal dispersion contains electrically charged particles whose interactions can strongly affect physical stability, rheological behavior, sedimentation, re-dispersion, and filtration. One of the most useful parameters to understand such interactions is the *ζ-*potential. In particular, variations of this value in the reconstituted dispersion can be attributed to particle aggregation, to the presence of an absorbed layer on the interface or also to the drug leakage, which is the most well-known drawback of the freeze-drying process of liposomes, mainly when hydrophilic cargoes, that do not interact with the bilayer, are considered. Drug leakage is mainly attributed to vesicle fusion and/or gel to liquid–crystalline phase transitions of the membrane lipids during drying with consequent loss of the encapsulated material. To control the drug leakage, liposomal cake is generally washed and the unentrapped drug is removed from the reconstituted dispersion through a proper purification method (e.g., column separation) prior of the assay of the drug still encapsulated in the vesicles [105]. This is of course one of the first controls to be carried out after freeze-drying to assure the loading of therapeutic dose.

## 7. Conclusion

After decades of studies in this field, no “rules of thumbs” can be proposed to make the design and development of a freeze-dried liposomal formulation easier. Indeed, it is not just a matter of the type and location (bulk water or vesicle inner core) of protectant or the definition of process parameters, but it is the intricate relation among such variables and bilayer composition which determines the success of lyophilization to stabilize liposomes in a dried state.

The current guidelines issued by the main regulatory agencies on the development of liposomal products [9,106] do not mention any tools to characterize the quality of the freeze-dried products. Nevertheless, since many efforts have been designed to develop dried liposomal dosage forms and few products have been already tested by clinical trials [5,6], there is an urgent need to release some specifics on the quality control of lyophilized liposomal products at the regulatory level. Recent advances in imaging techniques combined with the information related to the reconstituted suspensions could improve the know-how required not only to optimize the process, but also quality controls of lyophilized formulations at an industrial level.

## Figures and Tables

**Table 1:** Phase pre-transition (*T_p_*) and transition (*T_m_*) temperatures of fully hydrated phospholipids.

Phospholipids	Acyl Chains	*T_m_* (°C)	*T_p_* (°C)	Ref.
DLPC	12:0/12:0	−1	-	[12,13]
DMPC	14:0/14:0	24	22.0	[13]
DPPC	16:0/16:0	40.5	35.5	[14]
DSPC	18:0	49.1	54.5	[13]
DOPC	18:1	−18	9.0	[13]
HSPC	16:0/18:0	53.6	47.8	[15]
EPC	Mixed chains	−15 to −20	-	[13]

1,2-dilauroyl-sn-glycero-3-phosphocholine (DLPC); 1,2-dimyristoyl-sn-glycero-3-phosphocholine (DMPC); 1,2-dipalmitoyl-sn-glycero-3-phosphocholine (DPPC); 1,2-distearoyl-sn-glycero-3-phosphocholine (DSPC); 1,2-dioleoyl-sn-glycero-3-phosphocholine (DOPC); hydrogenated soy phosphocholine (HSEP); egg phosphocholine (EPC).

**Table 2:** Overview of the main excipients tested as protectants of liposomes upon freeze-drying. To allow a quick comparison of the data, it was arbitrarily assumed that the reconstitution is acceptable (Y) when a monodisperse population was obtained upon re-hydration and the hydrodynamic diameter of the resuspended liposomes was lower than 3 times with respect to the value of the not freeze-dried formulation. Leakage was considered avoided (effective) if the retention of the entrapped substance after freeze-drying was at least 90% of the original value.

Protectant	Liposome Components	Effect		Ref.
		Reconstitution (Protectant:lipids Ratio)	Avoiding Leakage (Substance)	
**Mannitol**	POPC or DSPC: ls-PG: DSPE-PEG_2000_	Y (5-10:1 *w*/*w*)	-	[23]
**Glucose**	POPC or DSPC: ls-PG: DSPE-PEG_2000_ DOPC or DPPC or EPC EPC	Y (5-10:1 *w*/*w*) - N ( (0-500 mg/mL)	- Ineffective (calcein) Ineffective in the range (CF)	[23]
				[60]
				[61]
**Lactose**	POPC or DSPC: ls-PG: DSPE-PEG_2000_	Y (5-10:1 *w*/*w*)	-	[23]
	DPPC:DPPG:CHOL	Y (5% *w*/*w* in; 15% out)	Effective (doxorubicin)	[48]
	SPC or SPC:SPS or SPC:SPS:CHOL	Y (5% *w*/*w*)	-	[52]
**Maltose**	DOPC or EPC DPPC	- -	Ineffective (calcein) Effective (calcein)	[60]
	DPPC	-	Effective ( > 5:1 mol/mol)	[62]
	DPPC:DPPG:CHOL	Y (5% *w*/*w* in; 15% out)	Effective (doxorubicin)	[48]
**Sucrose**	DPPC:DPPG:CHOL	Y (5% *w*/*w* in; 15% out)	Effective (doxorubicin)	[48]
	DOTAP:DOPE DOTAP:DOPE:DSPE-PEG	Y (57:1 *w*/*w*) Y (51:1 *w*/*w*)	- -	[63]
	EPC	Y (0-500 mg/mL)	≥50 mg/mL (CF)	[61]
	SPC:CHOL:DOTAP EPC:CHOL:DOTAP HSPC:CHOL:DOTAP	Y (3-15:1 *w*/*w*) Y (3-15:1 *w*/*w*) Y (20-25:1 *w*/*w*)	Ineffective (ATP) Ineffective (ATP) Not tested	[64]
	DPPC:CHOL:DSPE-PEG	Y (6:1 *w*/*w*)	Ineffective (prednisolone)	[65]
	DOTAP:CHOL	Y (1.6-2.7:1 *w*/*w*)	Ineffective (decapeptide)	[66]
	SPC SPC:SPS SPC:SPS:CHOL	Y (5% *w*/*w*) Y (5% *w*/*w*) Y (5% *w*/*w*)	- - -	[52]
**Trehalose**	DPPC EPC DSPC:DSPE-PEG_2000_:ls-PG POPC:DSPE-PEG_2000_:ls-PG DPPC:DPPG:CHOL DOTAP:DOPE DOTAP:DOPE:DSPE-PEG SPC or EPC:CHOL:DOTAP HSPC:CHOL:DOTAP DPPC:CHOL:DSPE-PEG DOTAP:CHOL EPC:CHOL	Y (> 0.5:1) Y (> 0.3:1 *w*/*w*)	Ineffective (CF) Ineffective (CF)	[53]
		Y (5-10:1 *w*/*w*) Y (5-10:1 *w*/*w*)	- -	[23]
		Y (5% *w*/*w* in; 15% out)	Effective (doxorubicin)	[48]
		Y (57:1 *w*/*w*) Y (51:1 *w*/*w*)	- -	[63]
		Y (5-15:1 *w*/*w*) Y (5-25:1 *w*/*w*)	Effective (< 10:1 *w*/*w*) (ATP)	[60]
		Y (6:1 *w*/*w*)	Ineffective (prednisolone)	[65]
		Y (1.6-3.7:1 *w*/*w*)	Ineffective (decapeptide)	[66]
		Y (2-8:1 mol/mol) N (10:1 mol/mol)	Effective at 4:1 mol:mol (ibuprofen)	[44]
**Maltotriose**	DOPC or EPC DPPC	- -	Ineffective (calcein) Effective (calcein)	[60]
**Maltotetraose**	DOPC or EPC DPPC	- -	Ineffective (calcein) Effective (calcein)	
**Maltoexaose**	DOPC or EPC DPPC	- -	Ineffective (calcein) Effective (calcein)	
**Maltoheptaose**	DOPC or EPC DPPC	- -	Ineffective (calcein) effective (calcein,>90%)	
	DPPC	-	Ineffective (calcein, 75%)	[62]
**HES**	EPC	Y (0-500 mg/mL)	Ineffective (CF)	[61]
**Dextran (1.5 kDa)**	DOTAP:DOPE DOTAP:DOPE:DSPE-PEG	Y (57:1 *w*/*w*) Y (51:1 *w*/*w*)	- -	[63]
**Dextran (5 kDa)**	DOTAP:DOPE	Y (57:1 *w*/*w*)	-	
**Dextran (40 kDa)**	DOTAP:DOPE:DSPE-PEG	Y (57:1 *w*/*w*)	-	
**Dextran (480 KDa)**	DPPC or EPC	Y (5-15:1 *w*/*w*)	Ineffective (CF)	[53]
**Inulin (1.8 kDa)**	DOTAP:DOPE DOTAP:DOPE:DSPE-PEG	Y (57:1 *w*/*w*) Y (51:1 *w*/*w*)	- -	[63]
**Inulin (4 kDa)**	DOTAP:DOPE DOTAP:DOPE:DSPE-PEG	Y (57:1 *w*/*w*) Y (51:1 *w*/*w*)	- -	[63]
**HP-β-cyclodextrin**	DPPC:CHOL:DSPE-PEG	Y (6:1 *w*/*w*)	Effective (prednisolone)	[65]
**Quercetin-3-O-glucoside**	EPC EPC:EPE EPC:DLPE	Y (30 mol %) N (30 mol %) Y (30 mol %)	Ineffective (CF)	[42]
**Quercetin-3-O-rhamnoside**	EPC EPC:EPE EPC:DLPE	Y (30 mol %) N (30 mol %) Y (30 mol %)	Ineffective (CF)	[42]
**Kaempferol-3-O--glucoside**	EPC or EPC:EPE or EPC:DLPE	Y (30 mol/mol)	Ineffective (CF)	[42]
**Kaempferol-7-O--glucoside**	EPC or EPC:EPE or EPC:DLPE	Y (30 mol/mol)	Ineffective (CF)	[42]
**Arginine**	EPC:CHOL	Y (4:1 mol/mol, only)	Effective (ibuprofen)	[44]
**Histidine**	EPC:CHOL	Y (4:1 mol/mol)	Effective (ibuprofen)	[44]
**Lysine**	EPC:CHOL	Y (2-4:1 mol/mol)	Effective (4:1 mol:mol) (ibuprofen)	[44]
**Gelatin**	SPC:CHOL	Y (5-20% *w*/*v* in)	Effective (>10%, 94.2% CF)	[67]

Abbreviations: POPC: 1-Palmitoyl-2-oleoyl-sn-glycero-3-phosphocholine; DSPC: 1,2-distearoyl-sn-glycero-3-phosphocholine; ls-PG: 1-stearoyl-2-hydroxy-sn-glycero-3-[phospho-rac-1-glycerol]; DSPE-PEG2000: 1,2-distearoyl-sn-glycero-3-Phosphoethanolamine-*N*-[methoxy (polyethylene glycol)-2000]; EPC: Egg yolk l-a-phosphatidylcholine; DOPC: Dioleoyl-t-c~-phosphatidylcholine; DPPC: dipalmitoyl-l-ce-phosphatidylcholine; DPPG: 1,2-dipalmitoyl-sn-glycero-3-phospho-(1′-rac-glycerol); CHOL: cholesterol; SPC: Soybean phosphatidylcholine; SPS: soybean phosphatidylserine; DOTAP: *N*-[1-(2,3-Dioleoyloxy)propyl]-*N*,*N*,*N*-trimethylammonium; DOPE: 1,2-dioleoyl-sn-glycero-3-phosphoethanolamine; CF: carboxyfluorescein; DLPE: 1,2-dilinoleoyl-sn-glycero-3-phosphoethanolamine; EPE: egg phosphatidylethanolamine; HSPC: l-α-phosphatidylcholine, hydrogenated (Soy); in/out: in, inside the liposome core; out, in the bulk medium. Y: Yes; N: No.

**Table 3:** Collapse temperature (*T_c_*), glass transition temperature of maximum freeze concentrate (*T_g_*’) and water content remaining within an amorphous phase (*W_g_*’) of various solutes (adapted from [75]).

Compound	*T_c_* (°C)	*T_g_*’ (°C)	*W_g_*’ (%)
Glucose	−40	−43	29.1
Fructose	−48	−42	49.0
Sorbitol	−27	−43	18.7
Inositol	−27		
Sucrose	−32	−32.0	35.9
Lactose	−32	−28.0	40.8
Maltose	−32	−29.5	20.0
Raffinose	−26	−26.5	
Threalose		−29.5	16.7
Dextran	−9	−9	
HPβCD (hydroxypropyl-β-cyclodextrin)	−8	−8	
Poly(vinyl pyrrolidone)	−23	−19.5	
Poly(ethylene glycol)	−13	−13	
